# An endometrial receptivity scoring system basing on the endometrial thickness, volume, echo, peristalsis, and blood flow evaluated by ultrasonography

**DOI:** 10.3389/fendo.2022.907874

**Published:** 2022-08-09

**Authors:** Chun-hui Zhang, Cheng Chen, Jia-rui Wang, Yue Wang, Si-xi Wen, Yan-pei Cao, Wei-ping Qian

**Affiliations:** ^1^ Center for Reproductive Medicine, Peking University Shenzhen Hospital, Shenzhen, China; ^2^ Department of Ultrasonography, Peking University Shenzhen Hospital, Shenzhen, China

**Keywords:** endometrial receptivity, three-dimensional ultrasound, endometrial thickness, endometrial volume, echo, endometrial peristalsis, blood flow

## Abstract

**Background:**

Establishing a successful pregnancy depends on the endometrium and the embryo. It is estimated that suboptimal endometrial receptivity account for one-third of implantation failures. Despite the indepth understanding of the processes associated with embryo-endometrial cross-talk, little progress has been achieved for diagnosis and treatments for suboptimal endometrial receptivity.

**Methods:**

This retrospective study included women undergoing their first frozen-thawed embryo transfer (FET) cycles at our reproductive medicine center from March 2021 to August 2021. Transvaginal three-dimensional (3D) ultrasound was performed in the morning on the day of embryo transfer for all the thawed embryo transfer patients, to evaluate endometrial receptivity, including endometrial thickness, echogenicity, volume, movement and blood flow.

**Results:**

A total number of 562 patients of FET with 315 pregnancies (56.0%) was analyzed. It was found that only the echo of the endometrial central line was different between the pregnant group and non-pregnant group. Other parameters, such as endometrial thickness, volume, endometrial peristalsis, or the endometrial blood flow were not statistically different between the two groups. Then, according to the relationship between the different groups and the clinical pregnancy rate, a score of 0 to 2 was respectively scored. The sum of the scores for the six items was the patient’s endometrial receptivity score. It showed that the clinical pregnancy rate increased as the endometrial receptivity score increased, and when the receptivity score reaches at least 5, the clinical pregnancy rate is significantly improved (63.7% versus 49.5%, *P*=0.001).

**Conclusion:**

We developed an endometrial receptivity scoring system and demonstrated its validity. It may aid clinicians in choosing the useful marker in clinical practice and for informing further research.

## Introduction

Successful embryo implantation requires embryos with developmental potential and receptive endometrium. Continuously mature embryo laboratory operation technology and embryo culture technology have significantly improved the quality of embryos. However, we know very little about the endometrial receptivity. In a natural menstrual cycle, the endometrium undergoes a series of dynamic changes, but why it can accept embryo implantation only during a short time window is something we need to work hard to explore. In the frozen-thawed embryo transfer (FET) cycle, especially in the presence of a high-quality embryo with developmental potential, a receptive endometrial environment is essential for successful embryo implantation. Endometrial receptivity refers to the ability of the endometrium to accept embryo adhesion, implantation, and subsequent development (1).

Because of its accuracy and non-invasiveness, transvaginal ultrasound is widely used in the field of assisted reproduction, not only for monitoring follicles, but also for evaluating endometrial receptivity (2). The ultrasonic markers for evaluating endometrial receptivity include endometrial thickness, volume, echo, peristalsis, blood flow, etc (3).

Endometrial thickness is one of the most widely used evaluation markers of endometrial receptivity (4). Studies have shown that the embryo implantation rate increases significantly when the endometrial thickness reaches 7 or 8mm (5), but there are also cases reported that embryo implantation occurs when the endometrial thickness is only 3.7mm (6). Secondly, the occurrence of ectopic pregnancy such as tubal pregnancy and cervical pregnancy also shows that the thickness of the endometrium is not a necessary condition for embryo implantation. Endometrial volume, a neglected ultrasound marker of endometrial receptivity, can be more comprehensive representation of the entire endometrium than the endometrial thickness of a particular section. In recent years, due to the development of ultrasound technology, more and more studies have been conducted on the relationship between endometrial volume and embryo implantation (7). It may be used as one of the markers to evaluate endometrial receptivity.

The echo of the endometrium with a triple line pattern can be used as one of the predictors of clinical pregnancy (8). However, some studies have reached inconsistent conclusions, which may be due to differences in trial design, different evaluation methods, different ovulation induction schemes or different evaluation time points (9).

The endometrium in which the embryo implanted was alive, not static. The inner part of the myometrium has been revealed to generate contractions controlled by estrogen and progesterone changes, causing the endometrium to generate wave-like or peristaltic movements (10). In general, the direction of endometrial movements is from the fundus to the cervix during the menstrual period, which facilitates the discharge of menstrual blood. With the increase of estrogen levels, the direction of endometrial movements is mainly from the cervix to the fundus in the middle of menstruation, which helps the transport of sperm. After ovulation, increased progesterone inhibits myometrium contraction. The reduced and irregular endometrial movements contribute to blastocyst implantation. The relationship between the frequency, direction, and amplitude of endometrial movements and pregnancy outcome needs to be further studied in the field of assisted reproduction.

More and more studies have shown that the blood flow of the endometrium is very important for embryo implantation (11, 12). Not only the blood flow in the endometrium, but also the blood flow under the endometrium (13). The blood supply of the endometrium comes from radial arteries, which pass through the myometrium-endometrial junction and divide into the basilar artery supplying the basal layer, and then form a spiral artery to the surface of the endometrium. At the junction of the myometrium and the endometrium, ultrasound shows a hypoechoic area, which is called the subendometrial area. Histological studies have confirmed that the subendometrial area close to the endometrium is the innermost layer of the myometrium (14). Compared with the outer myometrium, the muscle cells are tighter and the blood flow is more abundant. Three-dimensional Doppler ultrasound can be used to detect the blood flow of the endometrium and subendometrium. Studies have shown that abundant endometrial and subendometrial blood flow is closely associated with IVF pregnancy outcomes (15).

Endometrial receptivity enables the endometrium to provide an optimal environment for embryo implantation. Despite the indepth understanding of the processes associated with embryo-endometrial cross-talk, little progress has been achieved for its clinical integration in terms of diagnostic tests and treatments for suboptimal endometrial receptivity (16, 17). A single marker may be difficult to accurately reflect endometrial receptivity. Therefore, the purpose of this study was to analyze the association of various ultrasound parameters of endometrial receptivity, including endometrial thickness, volume, echo, movements and blood flow, with clinical pregnancy rates in the frozen-thawed embryo transfer cycle. In order to aid clinicians in choosing the useful marker in clinical practice and for informing further research, we developed a scoring system for endometrial receptivity.

## Material and methods

### Study design and participants

**Figure 1:**
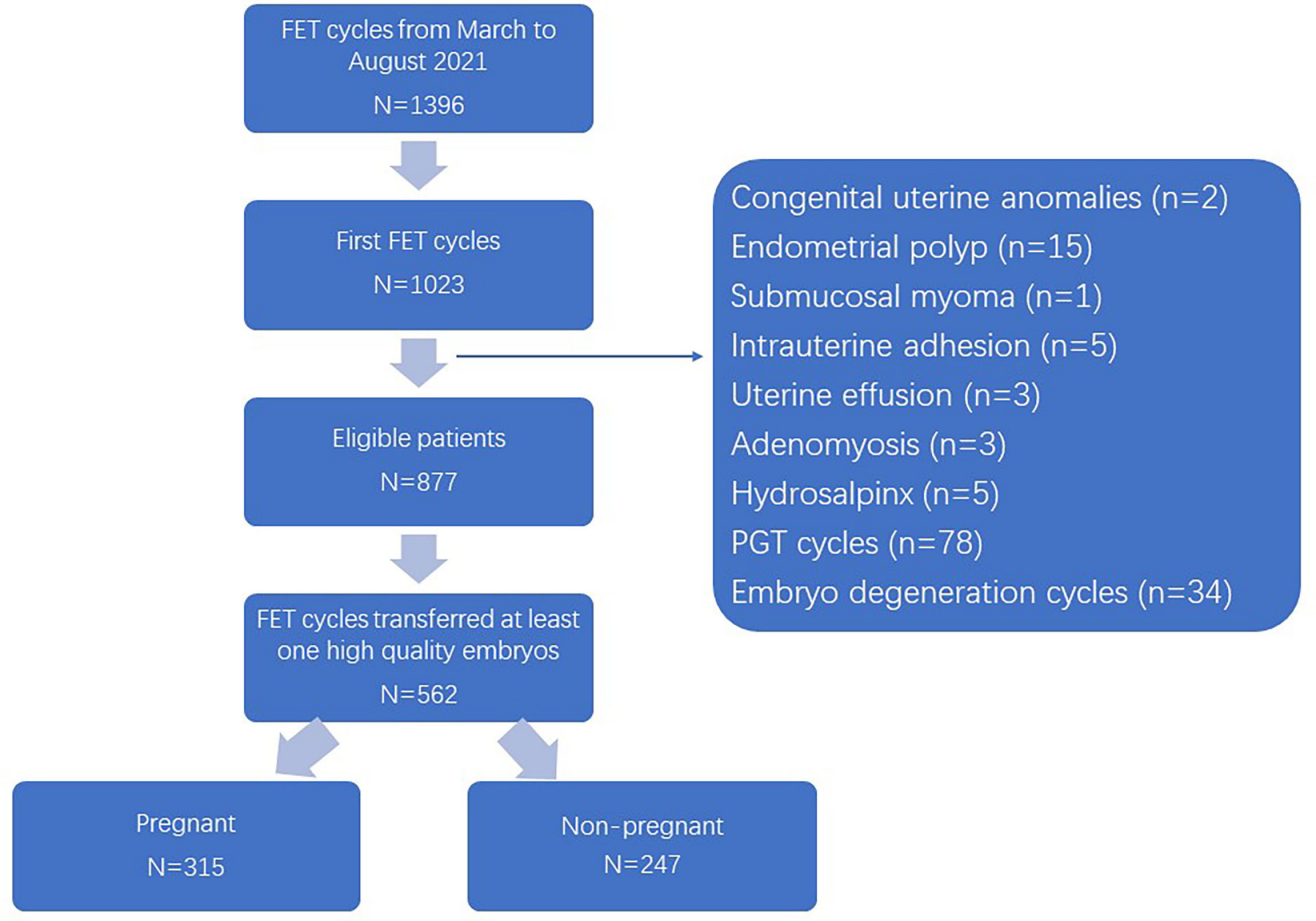
Flowchart of the included patients.

This retrospective study included 562 women undergoing their first FET cycles at Peking University Shenzhen Hospital during the duration from March 2021 to August 2021. Written informed consent to participate in this study was provided by all patients. The flowchart of the included patients was shown in **Figure 1**. Ethical review and approval for this study was obtained from the ethical committee of Peking University Shenzhen Hospital. We excluded patients if one of the following criteria was met (1): congenital uterine anomalies or acquired uterine diseases including endometrial polyp, submucosal myoma, intrauterine adhesion, uterine effusion and adenomyosis (2); hydrosalpinx (3); endometriosis (4); pre-implantation genetic test cycles (5); oocyte donation cycles (6); freeze-thaw embryo transfer cycles which all transferred embryos were non-high-quality embryos. That is to say, FET cycles with the transfer of at least one high-quality embryo were included in the analysis.

### Treatment protocol

Endometrial preparation protocols in our center are mainly included natural ovulatory cycles, ovulation induction cycles, and hormone replacement treatment (HRT) cycles based on the regularity of the menstrual cycle. For natural ovulatory cycles, transvaginal ultrasound monitoring of endometrial thickness and the diameter of the dominant follicle begins on the 10-12th day of the menstrual cycle. When the diameter of the dominant follicle reaches 18mm, a blood sample is obtained for luteinizing hormone (LH) levels. Also, transvaginal ultrasound was performed every other day, or daily if necessary, to determine the day of ovulation. The timing of embryo transfer was mainly determined by the day of ovulation. Thawing and transferring of cleavage embryos are on the 3rd day after ovulation, and transferring of blastocysts are on the 5th day after ovulation. A maximum of two embryos can be transferred. Intramuscular injection of a daily dose of 40mg progesterone in oil (progesterone injection, Baiyunshan, China) was started from the day of ovulation, and then convert to oral dydrogesterone at a dose of 10mg (Duphaston, Abbott, OLST, The Netherlands) twice a day and vaginal progesterone gel (Crinone, Merck Serono, Watford, UK) at a dose of 90mg once a day after embryo transferred. All the luteal support regimens were continued until 8 weeks of gestation, and then gradually reduced the dose. For the ovulation induction cycles, oral letrozole at a dose of 2.5-5 mg/d (Letrozol Tablets, Hengrui Pharmaceutical Co., Ltd, Jiangsu, China) once a day for 5 days from the 2-5 days of the menstrual cycle. Transvaginal ultrasound was performed to monitor the diameter of the dominant follicle from the 10-12th day of the menstrual cycle. If no dominant follicle develops, human menopausal gonadotropin (HMG, 75IU, Livzon Pharmaceutical Factory, China) 75-150IU can be injected intramuscularly every day or every other day. When the diameter of the dominant follicle reaches 18mm, a blood sample is obtained for LH levels. If the LH peak appears, wait for natural ovulation, or give urinary human chorionic gonadotropin (HCG, 5,000IU per ampoule, Profasi; Serono) at a dose of 6000-10000IU to induce ovulation. The embryo transfer time and the luteal support regimens are consistent with the natural cycle. For HRT cycles, oral estrogen (progynova, Bayer, Leverkusen, Germany) at a dose of 4-6mg/d was commenced on the second or third day of the menstrual cycle, transvaginal ultrasound examination was performed to measure endometrial thickness and to confirm that no dominant follicle had emerged. When the endometrial thickness reached 8mm, intramuscular injection of 40mg/d progesterone in oil was administered and embryo thawing and transfer was planned. If the endometrial thickness is less than 8mm, the dose of progynova can be increased to 8mg/d or other types of estrogen can be used. The day of progesterone administrated was defined as D0, cleavage embryo transferred on D3 and blastocyst transferred on D5. The luteal support regimens are consistent with the natural cycle. The use of estrogen was gradually reduced after ultrasound determination of gestational sac and fetal heart. The gonadotropin releasing hormone agonist (GnRHa, 3.75mg triptorelin for injection, Ferring, Switzerland) HRT cycle is to use GnRHa 3.75mg on the second day of the menstruation, and the subsequent medication is the same as the HRT cycles after 1-2 cycles of down-regulation.

According to the ASEBIR consensus (18), high-quality embryos are defined as cleavage-stage embryos with blastomeres ≥ 7, fragmentation ≤ 10%, uniform blastomeres, no vacuoles, and normal zona pellucida. High-quality blastocysts are grade 3BB and above. The embryos had been cryopreserved according to the vitrification protocol. All embryo transfers were performed using Cook catheter (Gyn, Spencer, IN, USA) under ultrasound guiding.

### Endometrium measurement

Transvaginal three-dimensional (3D) ultrasound was performed in the morning on the day of embryo transfer for all the first-cycle thawed embryo transfer patients, to evaluate endometrial receptivity, including endometrial thickness, echogenicity, volume, movement and blood flow. All ultrasound parameters were examined by the same doctor using the same ultrasound instrument. Endometrial thickness refers to the distance from the interface between the endometrium and myometrium on one side to the interface of the endometrium and myometrium on the other side. Due to the effect of progesterone, the endometrial echo is mainly hyperechoic. Ultrasound mainly distinguishes whether the echo of the endometrial functional layer was uniform and whether the endometrial central echogenic line was clear. Observe and record the movement of the endometrium within 5 minutes, including the presence or absence of endometrial peristalsis and the direction of movement, and then review it in fast forward mode and normal mode. The direction from the cervix to the fundus was defined as positive peristalsis. The direction from the fundus to the cervix was defined as negative peristalsis. The direction of the endometrium was unclear, that is to say, although it is moving, it cannot clearly distinguish whether it was positive or negative, which was defined as non-directional. The ultrasound machine was switched to the 3D mode with power Doppler. The area of interest was the longitudinal view of the uterus. The pulse repetition frequency was chosen for a color velocity range of 3cm/s, and the color gain was adjusted to 80% ± 2% to optimize the detection of blood flow in the small vessels. Identical color Doppler settings were used in all patients to standardize the examination. According to the depth of blood flow, they are divided into four groups: no blood flow, no more than 1/2 of the functional layer, more than 1/2 of the functional layer, and blood flow reaching the endometrial surface. The setting for this study was: frequency mid; dynamic set 2; power Doppler map 5. The sector of interest covering the endometrial cavity in a longitudinal plane of the uterus was adjusted, and the sweep angle was set to 90 to ensure that a complete uterine volume was obtained. 3D volume was acquired keeping the patient and the 3D transvaginal probe still during the volume acquisition. Stored volumes were analyzed by a single observer with VOCAL software.

### Outcome measures

The primary outcome of the study was the clinical pregnancy rate. Biochemical pregnancy was defined as positive β-hCG 14 days after embryo transfer and no gestational sac could be seen two weeks later. Clinical pregnancy was defined as ultrasound confirmation of gestational sac and fetal heart rate four weeks after embryo transfer. Ongoing pregnancy was defined as pregnancy with a detectable heart rate after 12 weeks of gestation. Miscarriage was defined as spontaneous pregnancy loss after a clinical pregnancy with a gestational sac visible in the uterine cavity. Ectopic pregnancy was defined as extra-uterine visualization of the gestation sac.

### Statistical analysis

The quantitative variables were tested for the normality of the distribution using the Kolmogorov-Smirnov test. Data were expressed as means ± standard deviation or median (interquartile ranges) according to the normality. Statistical comparison was carried out by Students’s t-test. The qualitative variables were represented as frequencies and percentages. Differences in these measures between the study groups were accessed by chi-square analysis or Fisher’s exact test if expected frequencies were less than five. All statistical calculations were performed by SPSS software (Statistical Package for the Social Sciences Version 19.0). P<0.05 was considered statistically significant.

## Results

A total of 562 patients who underwent frozen-thawed embryo transfer in our reproductive medicine center from March to August 2021 were included. The total clinical pregnancy rate was 56.0% (315/562), the biochemical pregnancy rate was 7.1% (40/562), the twin pregnancy rate was 9.8% (55/562), the ectopic pregnancy rate was 0.5% (3/562), the spontaneous abortion rate was 7.0% (22/315).

All included patients were divided into pregnant group and non-pregnant group according to whether clinical pregnancy was achieved. The baseline characteristics of the two groups were compared in **Table 1**. The age of the pregnant group (32.64 ± 3.92) was significantly younger than that of the non-pregnant group (34.82 ± 5.03). The basal E2 level was significantly lower than that of the non-pregnant group. The AMH level was significantly higher than that of the non-pregnant group. The duration of infertility years in the pregnancy group was shorter (3.40 ± 2.10 VS 4.07 ± 2.90). Other indicators, such as BMI, basal FSH levels, basal LH levels, endometrial preparation regimen, type of infertility, or infertility diagnosis, were not significantly different between the two groups.

**Table 1:** Baseline characteristics in women according to whether clinical pregnancy was achieved.

	Pregnant	Non-pregnant	*P* value
No. of cycles (n)	315	247	
Age (years)	32.64 ± 3.92	34.82 ± 5.03	0.000*
BMI (Kg/m^2^)	21.20 ± 2.74	21.35 ± 2.94	0.551
Baseline FSH (IU/L)	7.60 ± 3.10	8.15 ± 4.19	0.084
Baseline LH (IU/L)	5.51 ± 3.15	5.13 ± 3.58	0.186
Baseline E2 (pg/m))	39.00 ± 20.91	43.25 ± 24.47	0.030*
AMH (ng/ml)	5.19 ± 4.05	4.28 ± 3.00	0.003*
Regimen of endometrial preparation, n (%)			0.077
Natural cycle	51(16.2%)	55 (22.3%)	
Ovulation induction cycle	83 (26.3%)	66 (26.7%)	
HRT cycle	144 (45.7%)	110 (44.5%)	
GnRHa-HRT cycle	537(11.7%)	16 (6.5%)	
Type of infertility			0.679
Primary	124 (39.4%)	93 (37.7%)	
Secondary	191 (60.6%)	154 (62.3%)	
Duration of infertility (years)	3.40 ± 2.10	4.07 ± 2.90	0.002*
Infertility diagnosis, n (%)			0.170
Tubal factor	116 (36.8%)	75 (30.4%)	
Ovulation disorder	31 (9.8%)	19 (7.7%)	
Endometriosis	15 (4.8%)	13 (5.3%)	
Diminished ovarian reserve	9 (2.9%)	15 (6.1%)	
Male factor	43 (13.7)	28 (11.3%)	
Unexplained and other	50 (15.9)	55 (22.3%)	
Multiple factors	50 (15.9%)	42 (17.0%)	

Date are presented as mean ± standard deviation or n (%). BMI = body mass index; FET = frozen-thawed embryo transfer; FSH = follicle stimulating hormone; LH = luteinizing hormone; E2 = estradiol; AMH = anti-mullerian hormone; HRT = hormone replacement therapy; GnRHa = gonadotrophin-releasing hormone agonist. *Refers to statistical difference.

When comparing the ultrasound parameters of endometrial receptivity between the pregnant group and the non-pregnant group, it was found that only the echo of the endometrial central line was different between the two groups. The echo of the endometrial central line in the pregnant group was clearer than that in the non-pregnant group (**Table 2**). The echogenicity of the endometrial functional layer in the pregnancy group was more homogeneous, but it did not reach statistical significance. Other parameters, such as endometrial thickness, volume, the presence or absence of endometrial peristalsis, the direction of endometrial peristalsis, or the endometrial blood flow were not statistically different between the two groups.

**Table 2:** Comparison of ultrasound parameters of endometrial receptivity between pregnant group and non-pregnant group.

	Pregnant	Non-pregnant	*P* value
No. of cycles (n)	315	247	
Endometrial thickness (mm)	10.72 ± 2.54	10.40 ± 2.72	0.142
Endometrial volume (ml)	4.67 ± 1.91	4.48 ± 1.79	0.224
Echo of the functional layer of endometrium, n (%)			0.084
Homogeneous	283 (89.8%)	210 (85.0%)	
Heterogeneous	32 (10.2%)	37 (15.0%)	
Endometrial central echogenic line			0.006*
Present	210 (66.7%)	136 (55.3%)	
Absent	105 (33.3%)	110 (44.7%)	
Endometrial peristalsis			0.468
Absent	124 (39.5%)	114 (46.2%)	
Positive	62 (19.7%)	48 (19.4%)	
Negative	44 (14.0%)	26 (10.5%)	
Both positive and negative	33 (10.5%)	26 (10.5%)	
Non-directional	51 (16.2%)	33 (13.4%)	
Endometrial blood flow			0.355
Absent	84 (26.8%)	74 (30.1%)	
No more than 1/2	69 (22.0%)	62 (25.2%)	
More than 1/2	77 (24.5%)	59 (24.0%)	
Reaching the endometrial surface	84 (26.8%)	51 (20.7%)	

Date are presented as mean ± standard deviation or n (%).*Refers to statistical difference.

### Endometrial receptivity scoring system

**Figure 2:**
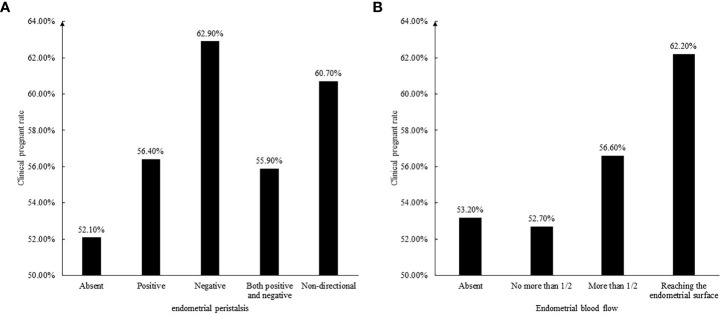
**(A)** The relationship between endometrial peristalsis and clinical pregnant rate. **(B)** The relationship between endometrial blood flow and clinical pregnant rate.

Since a single ultrasound marker may not fully reflect the receptivity of the endometrium, we assessed endometrial receptivity by considering the thickness, volume, echo, peristalsis, and blood flow of the endometrium. First, according to the thickness of the endometrium, it was divided into three groups: <8mm, 8-14mm and >14mm. The pregnancy rates of the three groups were 46.6%, 57.3%, and 61.5%, respectively. Therefore, the receptivity score was 0, 1, and 2. Similarly, according to the endometrial volume, it was divided into three groups: <3ml, 3-6ml, and >6ml, and the pregnancy rates of the three groups were 52.7%, 56.4%, and 57.8%, respectively. Because the pregnancy rates in the latter two groups were similar, it can be considered to divide into two groups with a cutoff of 3ml, and the receptivity score is 0 and 1 respectively. The clinical pregnancy rate was higher in patients with homogeneous echogenicity of the functional layer of endometrium, therefore, the score was 1 and the score was 0 in patients with heterogeneous echogenicity. Likewise, those with a clear endometrial central echogenic line were given a score of 1, and those with unclear endometrial central echogenic line were given a score of 0. The effect of endometrial peristalsis and blood flow on pregnancy rate is shown in **Figure 2**, so the endometrial receptivity score is shown in **Table 3**.

**Table 3:** Endometrial receptivity scoring system.

Endometrial receptivity scoring system			
Score	0	1	2
Endometrial thickness	<8 mm	8-14 mm	>14 mm
Endometrial volume	<3 ml	>3 ml	
Echo of the functional layer of endometrium	Heterogeneous	Homogeneous	
Endometrial central echogenic line	Absent	Present	
Endometrial peristalsis	Absent, Positive, Both positive and negaive	Non-directional	Negative
Endometrial blood flow	Absent, No more than 1/2	More than 1/2	Reaching the endometrial surface

**Figure 3:**
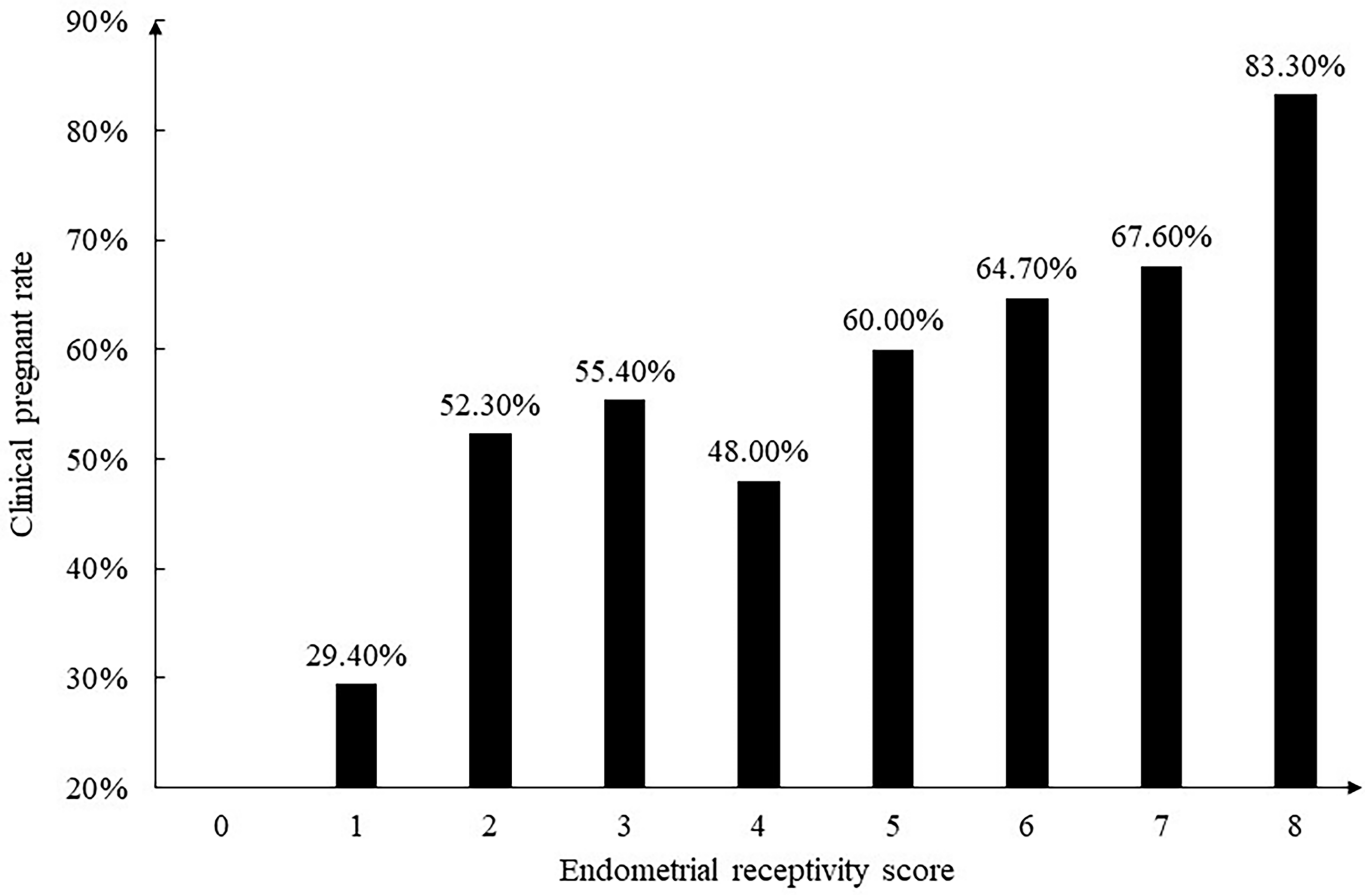
The relationship between endometrial receptivity score and clinical pregnant rate.

The receptivity scores of the pregnant group and the non-pregnant group were 4.60 ± 1.55 and 4.11 ± 1.53, respectively, and the difference was statistically significant (*P*=0.000). The pregnancy rates for different score groups are shown in **Figure 3**. It can be seen that the clinical pregnancy rate increased as the endometrial receptivity score increased, and when the receptivity score reaches at least 5, the clinical pregnancy rate is significantly improved (63.7% versus 49.5%, *P*=0.001).

After summarizing this scoring system, we included another 602 patients from September 2021 to April 2022 to further verify its validity. The results reached the same conclusion that the clinical pregnancy rate increased as the endometrial receptivity score increased. Similarly, we applied this endometrial receptivity scoring system to patients with non-high-quality embryos transferred during the same period and found that the clinical pregnancy rate was significantly improved when the endometrial receptivity score was greater than or equal to 5 (45.3% versus 33.7%, *P*=0.037).

## Discussion

Successful embryo implantation involves complex interactions between the embryo and the endometrium. Suboptimal endometrial receptivity account for almost one-third of implantation failures. Especially when we have high-quality embryos with developmental potential, how to achieve clinical pregnancy by improving endometrial receptivity is crucial. Endometrial receptivity have been the focus of extensive research for decades, since Rock *et al.* described the histological changes of the endometrium in 1937 (19). Recent researches identified a large variety of endometrial receptivity markers associated with clinical pregnant outcomes (3). The markers were evaluated by ultrasound, endometrial biopsy, hysteroscopy and endometrial fluid aspirate. Among them, the most famous molecular diagnostic tool was the endometrial receptivity array (ERA), which was proposed by Diaz-Gimeno *et al.* in 2011 (20). Ruiz-Alonso *et al.* assessed the endometrial receptivity by ERA in 85 women and found a higher rate of non-receptive endometrium in women with recurrent implantation failure compared to women without recurrent implantation failure (21). So, women with recurrent implantation failure may achieve pregnancy by undergo personalized embryo transfer (pET) according to the receptive status as identified by ERA. Because it is expensive, invasive and take a long time to report, it is not suitable for all embryo transfer patients. The aim of our study was to identify a simple scoring system for endometrial receptivity suitable for all embryo transfer patients, as is the case with ultrasound parameters. The reasons why we chose markers evaluated by ultrasound to develop the endometrial receptivity scoring system are as follows: First, in recent years, 3D color Doppler ultrasound technology has developed rapidly, with higher and higher resolution, and we can also measure parameters that could not be measured before. Second, transvaginal ultrasound is non-invasive. The ultrasound measurement takes a short time, and embryo transfer can be performed in the measurement cycle. Third, ultrasound measurement is simple and inexpensive, making it easy to generalize.

The endometrial receptivity markers evaluated by ultrasound included endometrial thickness, endometrial echo, endometrial wave-like activity and endometrial blood flow. Endometrial thickness was one of the most commonly investigated markers evaluated by ultrasound. In the present study, there was no significant difference in endometrial thickness between the pregnant group and the non-pregnant group (10.72 ± 2.54 VS 10.40 ± 2.72), which may be due to the fact that the current routine of our reproductive center was not to perform embryo transfer when the endometrial thickness was less than 8mm in the frozen-thawed embryo transfer cycles. Analysis of the single index of endometrial thickness found that the 90th percentile was 14mm. Therefore, in the endometrial receptivity scoring system, we divided the endometrial thickness into three groups, and the pregnancy rates were 46.6%, 57.3% and 61.5%, respectively. It can be seen that with the increase of endometrial thickness, the clinical pregnancy rate also has an upward trend. Other studies have also shown that after excluding endometrial polyps or other endometrial hyperplasia lesions, the increase in endometrial thickness alone does not affect the clinical pregnancy rate (22). However, Elnur *et al.* reported any significant relationship between baseline endometrial thickness (Day 3 of cycle) or endometrial thickness change (from baseline to start of progesterone supplementation) and clinical pregnancy rates in frozen embryo transfer cycles (23). Bahar *et al.* found that the endometrial thickness was similar between women who achieved a live birth and those who did not after fresh or frozen-thawed embryo transfer. There was no linear association between endometrial thickness and live birth or miscarriage rates, even without a cutoff. So it concluded that embryo transfer should not be denied when intracavitary pathology and inadvertent progesterone exposure were excluded, but only thinner endometrial thickness (24).

With the advancement of ultrasound detection technology, we can use 3D ultrasound to measure the volume of the endometrium. Compared with the endometrial thickness of a single plane, the endometrial volume can better reflect the overall state of the endometrium. One study reported clinical pregnant outcome based on the cut-off of 3.2ml for endometrial volume as measured on the day of the embryo transfer in the frozen-thawed embryo transfer cycle and the sensitivity was 80% and the specificity was 77.1% (25). In our study, there was only a modest increase in clinical pregnancy rate when the endometrial volume exceeded 6ml. Therefore, we considered 3ml as the cut-off value of endometrial volume and divided it into two groups with scores of 0 and 1 respectively. Ahmed *et al.* reported that endometrial volume can be a candidate predictor of *in-vitro* fertilization success to replace the traditional endometrial thickness, because it was significantly differenced between pregnant and non-pregnant women on day of triggering and embryo transfer (26). On the contrary, Assen *et al.* analyzed 142 patients in their study and found that endometrial volume assessed by 3D transvaginal ultrasound was not a useful tool for predicting pregnancy in single blastocyst embryo transfer cycles and the AUC was 0.48 (27).

Most studies showed that triple line pattern of endometrium was associated with higher clinical pregnancy rates. Their detection time points were at the stage of follicular growth and development, the day of HCG administration and the day before commencing progesterone. However, our detection time point was on the day before or on the day of embryo transfer, that is, after progesterone action, and therefore, the triple line endometrial pattern is rarely seen. Therefore, we simply divided the endometrial echo into whether the echo of the endometrial functional layer was uniform and whether the echo of the endometrial central line was clear. Yes or no, the scores are 1 and 0, respectively. The clinical pregnancy outcome also confirmed that the patients with clear endometrial central echogenic line had a significantly higher clinical pregnancy rate than its opposite (60.7% VS 48.8%, *P*=0.006). The clinical pregnancy rate was also higher in patients with homogeneous echo in the endometrial functional layer than its opposite, but it did not reach statistical difference (57.4% VS 46.4%, *P*=0.084).

When we observed the endometrium under ultrasound for 3 to 5 minutes, we will find that the endometrium is moving, not static. We call this endometrial peristalsis. Ijland et al. first recorded that women who conceived had lower endometrial peristalsis compared to women who never conceived (28). Swierkowski-Blanchard *et al.* found that women with clinical pregnancy following IUI were more likely to have low frequency endometrial peristalsis compared to women who failed to conceive (29). It seems like that endometrial peristalsis was unfavorable for embryo implantation. Chung *et al.* found that only the endometrial peristalsis frequency measured five minutes after the embryo transfer was reduced in women who achieved a clinical pregnancy compared to women who were not pregnant. The endometrial peristalsis acquired five minutes before and 60 minutes after the embryo transfer did not reach statistical difference (30). In our study, the presence or absence of endometrial peristalsis was not associated with clinical pregnancy rate (58.4% VS 52.7%, *P*=0.175), while the direction of endometrial peristalsis was associated with clinical pregnancy rate. The negative peristalsis group (fundus to cervix direction) had the highest clinical pregnancy rate, followed by the non-directional peristalsis group (peristalsis was existed, but the direction was not clear), and the others were lower. One of the reasons for the highest clinical pregnancy rate in the negative peristalsis group may be that at the time of embryo transfer, our clinicians placed the embryos close to the uterine fundus, and the negative peristaltic endometrium pushed the embryos a little towards the cervix. Really reached the best embryo implantation position.

Endometrial blood flow is essential to embryo implantation. Measurement of endometrial blood flow using 3D power Doppler in the field of reproductive medicine and their role in predicting IVF cycle outcome has attract a lot of attention across the world in recent years. Most studies suggested that blood flow to the endometrium helps improve clinical pregnancy rates (31). As in our findings, the clinical pregnancy rate in the group with endometrial blood flow was higher than that in the group without blood flow (57.2% VS 53.1%, *P*=0.378). although there was no statistical difference, the clinical pregnancy rate gradually increased with the deepening of blood flow (the depth of blood flow to the endometrial functional layer and whether the blood flow reaches the endometrial surface). Jianing *et al.* conducted a mate-analysis to evaluate the association between endometrial vasculature. They concluded that the endometrial vascularization index, flow index, and vascularization-flow index could help identify appropriate timing for frozen embryo transfer (32). Yuezhi *et al.* speculated that fertility stress might affect pregnancy outcomes by reducing endometrial and subendometrial blood flow (33).

It is very prescient that Applebaum proposed the uterine scoring system for reproduction (USSR) which comprises endometrial thickness, endometrial layering, myometrial contractions, myometrial echogenicity, uterine artery Doppler flow evaluation, endometrial blood flow and myometrial blood flow seen on gray-scale examination in 1995 (34). Later studies conducted by Mohd Shoeb Khan *et al.* and Ricardo *et al.* have demonstrated its clinical application value (35, 36). However, he did not specify how each score was derived. Second, the Applebaum Uterine Scoring System focused on the state of the entire uterus, including the echogenicity and blood flow of the myometrium, and the pulsatility index (PI) of the uterine arteries. Instead, we focused more closely on the shape and function of the endometrium. In recent years, with the development of ultrasound technology, the resolution has become higher and higher, and the state of the endometrium can be observed more clearly. The emergence of 3D technology makes it possible to measure the volume of the endometrium. It can be said that our research is the inheritance and update on the basis of previous research.

### Strengths

This is the first article to have proposed a simple scorning system for endometrial receptivity, which is not based on a single marker, but a comprehensive consideration of multiple markers detected by ultrasound, which is more reliable. The included markers include endometrial thickness, volume, echo, peristalsis, and blood flow evaluated by ultrasound, which have been studied extensively in recent years. It has been verified that the higher the score, the higher the clinical pregnancy rate. When the endometrial receptivity score is greater than or equal to 5, the clinical pregnancy rate exceeds 60% in the frozen-thawed embryo transfer cycle.

### Limitations

In the absence of data of live birth rate, we considered clinical pregnancy rate as a proxy outcome to confirm receptive endometrium. There were differences in baseline data between the pregnant and non-pregnant groups. The age of the pregnant group was younger than that of the non-pregnant group. The AMH level was higher than that of the non-pregnant group. The duration of infertility years in the pregnancy group were shorter. This may underestimate the accuracy of the ultrasound markers we evaluated. Because the absence of a clinical pregnancy may be a consequence of embryo quality (poor implantation potential or aneuploidy) or other factors (for example, age, abnormal endometrial microbiome (37) or systemic maternal conditions). It was a real-world retrospective study and the data and outcomes were from real patients in our reproductive center. Secondly, the sample size of the study was relatively small, and it is necessary to further expand the sample size to verify our conclusions.

## Conclusion

We developed an endometrial receptivity scoring system and demonstrated its validity. It may aid clinicians in choosing the useful marker in clinical practice and for informing further research.

## Data availability statement

The original contributions presented in the study are included in the article/supplementary material. Further inquiries can be directed to the corresponding author.

## Ethics statement

The studies involving human participants were reviewed and approved by the ethical committee of Peking University Shenzhen Hospital. The patients/participants provided their written informed consent to participate in this study.

## Author contributions

C-hZ, CC, and J-rW contributed equally to this work and co-first authors of the article. CC performed the analysis of data and wrote the manuscript. C-hZ and J-rW provided feedback. C-hZ, YW, and W-pQ designed the study and funding acquisition. All authors contributed to the article and approved the submitted version.

## Funding

This study was supported by the Project for Exploration of New method of Noninvasive Fertility Evaluation and Establishment of National Standard (2018YFC1002104) funded by the Ministry of Science and Technology of the People’s Republic of China, and the Project for Research Team of Female Reproductive Health and Fertility Preservation (SZSM201612065) funded by the Science, Technology and Innovation Commission of Shenzhen Municipality, the Project of basic research plan of Shenzhen Science and technology innovation Commission (JCYJ20200109140623124), and the Project of the Shenzhen High-level Hospital Construction Fund.

## Conflict of interest

The authors declare that the research was conducted in the absence of any commercial of financial relationships that could be construed as a potential conflict of interest.

## Publisher’s note

All claims expressed in this article are solely those of the authors and do not necessarily represent those of their affiliated organizations, or those of the publisher, the editors and the reviewers. Any product that may be evaluated in this article, or claim that may be made by its manufacturer, is not guaranteed or endorsed by the publisher.
